# Association between non-alcoholic fatty liver disease and coronary calcification depending on sex and obesity

**DOI:** 10.1038/s41598-020-57894-y

**Published:** 2020-01-23

**Authors:** Seok-hyung Kim, Hae Yeul Park, Hye Sun Lee, Kwon Soo Jung, Moon Hyoung Lee, Jong Hyun Jhee, Tae Hoon Kim, Jung Eun Lee, Hyung Jong Kim, Beom Seok Kim, Hyeong Cheon Park, Byoung Kwon Lee, Hoon Young Choi

**Affiliations:** 1 Department of Internal Medicine, Chuncheon Sacred Heart Hospital, Hallym University College of Medicine, Chuncheon-si, Gangwon-do 24253 Republic of Korea; 2 Department of Internal Medicine, Gangnam Severance Hospital, Yonsei University College of Medicine, Seoul, 06273 Republic of Korea; 3 Biostatics Collaboration Unit, Yonsei University College of Medicine, Seoul, 06273 Republic of Korea; 4 Department of Internal Medicine, Yongin Severance Hospital, Yonsei University College of Medicine, Yongin-si, Gyeonggi-do 17046 Republic of Korea; 5 Department of Internal Medicine, CHA Bundang Medical Center, CHA University, Seongnam-si, Gyeonggi-do 13496 Republic of Korea; 6 Department of Internal Medicine, Severance Hospital, Yonsei University College of Medicine, Seoul, 03722 Republic of Korea; 7 Severance Institute for Vascular and Metabolic Research, Yonsei University College of Medicine, Seoul, 03722 Republic of Korea

**Keywords:** Cardiology, Gastroenterology, Risk factors

## Abstract

Non-alcoholic fatty liver disease (NAFLD) is considered a hepatic manifestation of metabolic syndrome and is associated with cardiovascular outcomes. We investigated whether NAFLD was associated with coronary artery calcification (CAC) in participants without a previous history of cardiovascular disease and whether this association differed according to sex and obesity status after adjustment for other atherosclerosis risk factors, alcohol intake, and liver enzyme levels. Among 67,441 participants, data from 8,705 participants who underwent a fatty liver status and CAC assessment during routine health screening were analysed. CAC scores were calculated using computed tomography. NAFLD was diagnosed in patients with evidence of liver steatosis on ultrasonography. Obesity was defined as a body mass index of ≥25 kg/m^2^. Multivariate analysis showed a significant association between NAFLD and CAC in non-obese participants (odds ratio, 1.24 [95% confidence interval, 1.01–1.53]), whereas NAFLD and CAC were not associated in obese participants. Interaction analysis showed that the association between NAFLD and CAC was influenced by sex and obesity. Subgroup analysis revealed a significant association between NAFLD and CAC in non-obese male participants (odds ratio, 1.36 [1.07–1.75]), but not in female participants. Our study indicates that non-obese men with NAFLD are prone to CAC.

## Introduction

Cardiovascular disease (CVD) is a major cause of death, and its contribution to the overall disease burden is expected to increase. Therefore, there have been worldwide efforts to identify cardiovascular risk factors^1,2^.

Obesity is a well-established risk factor for CVD and mortality^3^; however, recent studies have reported the localized distribution of body fat rather than overall obesity^4,5^. In one study, regional fat distribution was shown to have a significant association with the risk of coronary heart disease after adjusting for body mass index (BMI)^4^.

Non-alcoholic fatty liver disease (NAFLD) can progress to liver cirrhosis and liver cancer, but its clinical manifestation is not confined to the liver. Moreover, it has been associated with an increased prevalence and incidence of CVD^6–8^. NAFLD is a highly prevalent metabolic abnormality closely linked to the overweight and obesity epidemic^8–10^. In tertiary liver centres, a majority of patients with NAFLD also have an increased BMI, but approximately 1 out of 8 NAFLD patients has normal BMI^11^. Globally, the prevalence of NAFLD in the non-obese population has been widely reported, ranging from 3% to 30%^12^.

Coronary artery calcification (CAC) is a non-invasive predictor of the burden of coronary atherosclerosis and is evaluated using computed tomography (CT). CAC scores have been reported to be associated with increased CVD events^13^. A previous study has shown that obesity is a risk factor for CAC irrespective of metabolic disorders^14^. Further, in a large health-screening population, the CAC score was significantly higher in subjects with NAFLD than in those with abdominal obesity after adjusting for risk factors for CVD and metabolic syndromes^5^. However, the association between NAFLD and CAC has not been consistently shown across all studies, which could be limited by the inadequate adjustment for established cardiovascular risk factors, selection bias, and study design^15–19^.

The present study aimed to investigate whether NAFLD was associated with CAC in participants without a previous history of CVD and whether this association differed according to obesity status after adjustment for other atherosclerosis risk factors, alcohol intake, and liver enzymes. We also evaluated the association between NAFLD and CAC in male and female participants.

## Methods

### Participants

**Figure 1:**
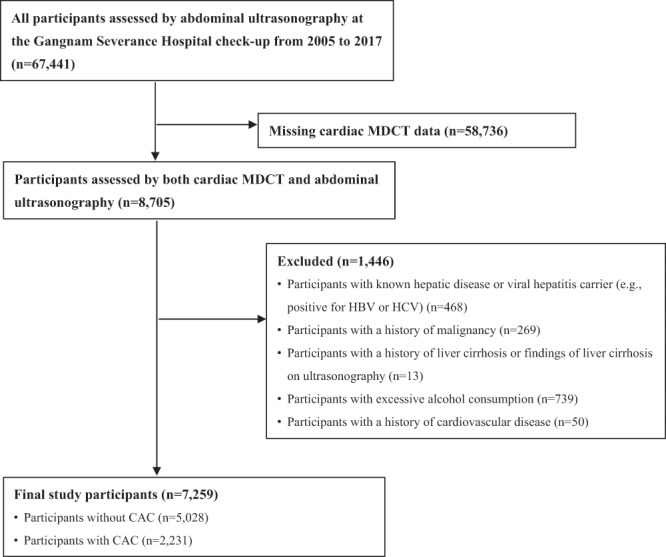
Diagram of participant enrolment. MDCT, multidetector computed tomography; HBV, hepatitis B virus; HCV, hepatitis C virus; CAC, coronary artery calcification.

This retrospective cross-sectional study included adult participants who voluntarily underwent health screening examination at Gangnam Severance Hospital from 2005 to 2017. Among 67,441 participants, data from 8,705 participants who had been evaluated for CAC and fatty liver status were collected from the medical records database. The study excluded an additional 1,446 participants who (1) had known hepatic disease or were positive for serologic markers of viral hepatitis B or C; (2) had excessive alcohol consumption (≥30 g/day for men and ≥20 g/day for women); (3) had a history of liver cirrhosis or findings of liver cirrhosis on ultrasonography; (4) had a history or current diagnosis of malignancy; and/or (5) had a history of CVD. Finally, 7,259 participants were included in this study (Fig. 1).

This study was performed in accordance with relevant guidelines and regulations. The Institutional Review Board of Gangnam Severance Hospital, Yonsei University College of Medicine, approved the study protocol (IRB No. 3-2019-0164) and the need for acquisition of informed consent from participants was waived owing to the retrospective nature of the study. The present study complied with the Guideline for Good Clinical Practice (GCP) by International Council for Harmonization of Technical Requirements for Registration of Pharmaceutical for Human use (ICH).

### Health screening examination

Health screening examination comprised initial consultation, anthropometric assessments, blood tests, and several imaging studies. Practice nurses collected and coded data on disease history, alcohol consumption, and smoking habits through initial consultation with participants. Irrespective of specific symptoms, abdominal ultrasound scan was included in all health screening programmes in order to detect the disease early. Health screening programmes, including CT coronary angiography, were conducted as a preventive measure to identify potential cardiovascular problems in voluntary participants regardless of heart-related symptoms.

### Clinical and laboratory data

We reviewed the participants’ medical records, including height, body weight, smoking status, alcohol intake, and blood pressure, which were measured during the visits. Blood samples were obtained on the day cardiac CT was performed after a fasting period of at least 12 hours. The following laboratory parameters were measured: C-reactive protein (CRP), creatinine, fasting glucose, aspartate aminotransferase (AST), alanine aminotransferase (ALT), gamma-glutamyl transpeptidase (γ-GT), and low-density lipoprotein (LDL)-cholesterol. LDL-cholesterol was measured directly with a homogenous enzymatic colorimetric assay using a Beckman Coulter AU5800 analyser (Beckman Coulter Inc., Brea CA, USA). BMI was calculated as body weight (kilograms) divided by the square of height (meters). Waist-hip-ratio was estimated based on the waist and hip circumference measured using bioelectrical impedance analysis (BIA, InBody570; InBody, Seoul, South Korea)^20^. Obesity was defined as a BMI of ≥25 kg/m^2^. Abdominal obesity was defined as a waist-hip ratio above 0.90 for men and above 0.85 for women. Obesity and abdominal obesity were defined according to the criteria given by the World Health Organization; particularly, the definition of obesity was based on specific criteria for Asians^21,22^. Smoking and alcohol consumption habits were determined from questionnaires. Participants were classified as current smokers, or ex-smokers or non-smokers based on their smoking habits. Average alcohol consumption per day was calculated using the frequency and amounts of beverages consumed per drinking day. Excessive alcohol consumption was defined as average alcohol intake ≥30 g/day for men and ≥20 g/day for women^23^. Patients were classified as drinkers (except those with excessive alcohol consumption) or non-drinkers on the basis of current alcohol intake. In this study, NAFLD was described as the presence of steatosis without secondary causes, such as hepatitis virus infection or excessive alcohol consumption^24^. Kidney function was assessed based on the estimated glomerular filtration rate (eGFR), which was calculated using the CKD-Epidemiology Collaboration equation and serum creatinine levels^25^.

### Assessment of coronary calcification and fatty liver status

Participants underwent cardiac multidetector CT (Philips Brilliance 64; Philips Medical Systems, Best, The Netherlands), with a 3-mm slice thickness and 1.5-mm reconstruction interval. Beta-blocking agents (25 mg atenolol) were administered to participants with a heart rate of >66 beats/min prior to CT. The CAC score was measured using the Agatston method^26,27^. CAC scores above zero indicated CAC^28^.

A diagnosis of fatty liver was based on abdominal ultrasonography using a 3.5-MHz transducer (HDI 5000; Philips, Bothell, USA). Ultrasonography was performed by four experienced radiologists who were unaware of the aims of the study and blinded to the laboratory findings. Hepatic steatosis was graded according to previously described criteria as follows: mild steatosis was recognized by slightly increased echogenicity in the liver parenchyma with relative maintenance of echogenicity in the walls of the portal veins and hepatorenal contrast; moderate steatosis was accompanied by a diffuse increase in parenchymal echogenicity with a loss of echogenicity in the wall of the portal veins, particularly from the peripheral branches; and severe steatosis was recognized by markedly increased echogenicity in the liver parenchyma with a loss of echogenicity in most of the portal vein wall, including the main branches, and a high hepatorenal contrast. In this study, any degree of steatosis in the liver was classified as NAFLD without secondary causes, such as hepatitis virus infection or excessive alcohol consumption^23,24^.

### Statistical analysis

Data are presented as median (25^th^ to 75^th^ percentile) for continuous variables or as frequency (percentage) for categorical variables. Statistical differences in clinical characteristics between the two groups were determined using the Mann-Whitney U test for continuous variables and the chi-square test for categorical variables. Univariate and multivariate logistic regression analyses with adjustment for the influence of confounders were performed to determine the association between CAC and NAFLD according to obesity status.

To test the combined effect of sex, obesity, and NAFLD, we tested their interactions with the interaction term for sex*obesity*fatty liver based on binary logistic regression models for outcome. The interaction between sex, obesity, and fatty liver was tested at a significance level of 0.15^29,30^.

Additional subgroup analyses were performed to identify the association between CAC and NAFLD, stratified by obesity status and sex after adjustment for multiple cofounders. Odds ratios (ORs) with 95% confidence intervals (CIs) were abstracted from the analysis and used to create a forest plot. Comparisons with *P*-values < 0.05 were considered statistically significant.

Mediation analyses were performed to evaluate the proportion of the total effect that could be explained by abdominal obesity. To test our main hypothesis, subjects were divided according to obesity or NAFLD status^31^. We assumed that all missing values were completely random and analysed the whole data, with the exception of these missing values. All analyses were conducted using the Statistical Package for the Social Sciences (SPSS) version 23.0 (IBM Corp., Armonk, NY, USA) and Statistic Analysis System (SAS) version 9.3 (SAS Inc., Cary, NC, USA).

## Results

### Baseline characteristics

The median age and BMI of the 7259 participants were 54 years and 24.1 kg/m^2^, respectively; 59.5% of the participants were males.

The participants were divided into two groups according to obesity status. Obese participants had a significantly higher prevalence of hypertension, diabetes, dyslipidaemia, and current smoking and current drinking status (*P* < 0.001, except diabetes, *P* = 0.018; current smoker, *P* = 0.005). Fasting glucose, LDL-cholesterol, CRP, triglyceride, AST, ALT, and γ-GT levels were significantly higher, whereas eGFR was lower in obese participants than in non-obese participants (*P* < 0.001). Obese participants had a significantly higher prevalence of NAFLD, abdominal obesity, and CAC than non-obese participants (Table 1).

**Table 1:** Characteristics of the study participants classified by obesity status.

	All participants (*n* = 7259)	Obese participants (*n* = 2839)	Non-obese participants (*n* = 4420)	*P-*value
Age (years)	54 (48–60)	54 (48–61)	54 (48–60)	0.051
Sex (male)	4309 (59.4)	2070 (72.9)	2239 (50.7)	<0.001
Hypertension (yes)	1462 (20.1)	812 (28.6)	650 (14.7)	<0.001
Diabetes (yes)	402 (5.5)	180 (6.3)	222 (5.0)	0.018
Dyslipidaemia (yes)	586 (8.1)	276 (9.7)	310 (7.0)	<0.001
Current smoker (yes)	1026 (19.8)	453 (21.7)	573 (18.5)	0.005
Alcohol status (yes)	3143 (43.3)	1376 (66.0)	1769 (56.8)	<0.001
BMI (kg/m^2^)	24.1 (22.0–26.3)	27.0 (25.9–28.8)	22.5 (20.9–23.8)	<0.001
SBP (mmHg)	125 (114–135)	130 (120–139)	121 (111–132)	<0.001
Fasting glucose (mg/dL)	97 (90–106)	100 (93–111)	95 (88–103)	<0.001
eGFR (mL/min/1.73 m^2^)	95 (84–103)	94 (82–102)	96 (85–104)	<0.001
LDL-cholesterol (mg/dL)	128 (106–153)	131 (108–158)	126 (104–149)	<0.001
CRP (mg/L)	0.7 (0.3–1.5)	1.0 (0.5–2.0)	0.5 (0.3–1.1)	<0.001
AST (IU/L)	24 (20–30)	26 (21–33)	23 (19–29)	<0.001
ALT (IU/L)	23 (16–32)	28 (20–40)	20 (15–28)	<0.001
γ-GT (IU/L)	24 (16–39)	31 (21–50)	20 (14–31)	<0.001
NAFLD (yes)	3328 (45.8)	1974 (69.5)	1354 (30.6)	<0.001
No steatosis	3931 (54.2)	864 (22.0)	3066 (78.0)	
Mild steatosis	1604 (22.1)	771 (48.1)	833 (51.9)	
Mild to moderate steatosis	284 (3.9)	164 (57.7)	120 (42.3)	
Moderate steatosis	1054 (14.5)	720 (68.3)	334 (31.7)	
Moderate to severe steatosis	171 (2.4)	131 (76.6)	40 (23.4)	
Severe steatosis	215 (2.9)	188 (87.4)	27 (12.6)	
Abdominal obesity (yes)	4698 (64.6)	2648 (93.3)	2050 (46.4)	<0.001
CAC (yes)	2231 (30.7)	1087 (38.3)	1144 (25.9)	<0.001

Values are expressed as number (%) for categorical variables or as median (25th to 75th percentile) for numeric variables, unless noted otherwise. BMI, body mass index; SBP, systolic blood pressure; eGFR, estimated glomerular filtration rate; LDL, low-density lipoprotein; CRP, C-reactive protein; AST, aspartate aminotransferase; ALT, alanine aminotransferase; γ-GT, gamma-glutamyl transpeptidase; NAFLD, non-alcoholic fatty liver disease; CAC, coronary artery calcification.

### Non-alcoholic fatty liver disease and coronary calcification

Univariate logistic regression analysis was performed to examine the association of individual covariates with CAC in order to identify risk factors for CAC. In all participants, all risk factors, including NAFLD, were significantly associated with CAC except LDL-cholesterol. In obese participants, increasing age (*P* < 0.001), male sex (*P* < 0.001), hypertension (*P* < 0.001), diabetes (*P* < 0.001), abdominal obesity (*P* < 0.001), LDL-cholesterol (*P* = 0.013), eGFR (*P* < 0.001), and AST (*P* < 0.001) significantly affected CAC. In non-obese participants, increasing age, male sex, hypertension, diabetes, abdominal obesity, eGFR (*P* < 0.001), CRP (*P* = 0.001), current smoking, AST, ALT, and γ-GT levels (*P* < 0.001) were significantly associated with CAC. NAFLD was significantly associated with CAC in both obese (*P* = 0.011) and non-obese (*P* < 0.001) participants (Supplementary Table S1).

Multivariate analysis was performed to investigate which variables were independently associated with CAC according to obesity status. Increasing age (*P* < 0.001), male sex (*P* < 0.001), hypertension (*P* < 0.001), diabetes (*P* < 0.001), obesity (*P* = 0.004), NAFLD (*P* = 0.001), current smoking (*P* = 0.011), and AST (*P* = 0.009) were independent predictors of CAC in all participants. Increasing age, male sex, hypertension, diabetes, and AST were independently associated with CAC irrespective of obesity. However, abdominal obesity was not independently associated with CAC in all participants and obese or non-obese participants (*P* = 0.428 and *P* = 0.216, respectively). NAFLD (*P* = 0.034) was independently associated with CAC only in non-obese participants (Table 2).

**Table 2:** Multivariate logistic regression analysis to determine risk factors affecting CAC.

Variables	All participants		Obese participants		Non-obese participants	
	OR (95% CI)	*P*-value	OR (95% CI)	*P*-value	OR (95% CI)	*P*-value
Age	1.11 (1.10–1.12)	<0.001	1.12 (1.10–1.13)	<0.001	1.11 (1.10–1.12)	<0.001
Male sex	3.24 (2.65–3.97)	<0.001	3.59 (2.67–4.83)	<0.001	2.88 (2.16–3.83)	<0.001
Hypertension	1.82 (1.57–2.11)	<0.001	1.65 (1.34–2.03)	<0.001	2.06 (1.66–2.56)	<0.001
Diabetes	1.82 (1.43–2.33)	<0.001	1.70 (1.19–2.44)	0.004	1.98 (1.42–2.77)	<0.001
Obesity	1.27 (1.08–1.49)	0.004				
Abdominal obesity	1.12 (0.90–1.40)	0.298	1.26 (0.71–2.26)	0.428	1.19 (0.91–1.55)	0.216
NAFLD	1.23 (1.06–1.44)	0.007	1.20 (0.95–1.52)	0.122	1.25 (1.02–1.54)	0.034
eGFR	1.00 (0.99–1.01)	0.836	1.01 (1.00–1.02)	0.150	0.99 (0.99–1.00)	0.091
CRP	1.00 (0.99–1.02)	0.904	1.00 (0.98–1.01)	0.905	1.01 (0.99–1.04)	0.397
Current smoking	1.27 (1.07–1.51)	0.008	1.14 (0.88–1.48)	0.310	1.44 (1.13–1.84)	0.004
Alcohol status	1.06 (0.90–1.24)	0.517	1.18 (0.92–1.51)	0.192	0.95 (0.77–1.19)	0.678
AST	1.01 (1.00–1.02)	0.006	1.02 (1.00–1.03)	0.013	1.01 (1.00–1.02)	0.049
ALT	1.00 (0.99–1.00)	0.252	1.00 (1.00–1.00)	0.482	0.99 (0.98–1.00)	0.083
γ-GT	1.00 (1.00–1.00)	0.077	1.00 (1.00–1.00)	0.798	1.00 (1.00–1.01)	0.027

CAC, coronary artery calcification; NAFLD, non-alcoholic fatty liver disease; eGFR, estimated glomerular filtration rate; CRP, C-reactive protein; AST, aspartate aminotransferase; ALT, alanine aminotransferase; γ-GT, gamma-glutamyl transpeptidase; OR, odds ratio; CI, confidence interval.

### Interaction effect and subgroup analysis

The three-way interaction effect, including sex*NAFLD, obesity status*NAFLD, and sex*obesity status*NAFLD, was obtained by analysing the effect of NAFLD and CAC according to sex and obesity status. All multinomial logistic regression models showed significant interactions for CAC (interaction *P* = 0.0210 before adjustment, *P* = 0.1291 in model 1, *P* < 0.001 in model 2, and *P* = 0.0002 in model 3), showing that the association between NAFLD and CAC was influenced by sex and obesity status (Table 3).

**Table 3:** Interaction test results with the interaction terms for sex, obesity status, and NAFLD via binary logistic regression models for CAC.

	Three-way interaction *P*-value
**Unadjusted**	0.021
**Adjusted**	
Model 1	0.129
Model 2	<0.001
Model 3	<0.001

Model 1, adjusted for age, sex, hypertension, diabetes, and abdominal obesity; model 2, adjusted for model 1 plus current smoking, estimated glomerular filtration rate, low-density lipoprotein, and C-reactive protein; model 3, adjusted for model 2 plus aspartate aminotransferase, alanine aminotransferase, gamma-glutamyl transpeptidase, and alcohol status.

NAFLD, non-alcoholic fatty liver disease; CAC, coronary artery calcification.

**Figure 2:**
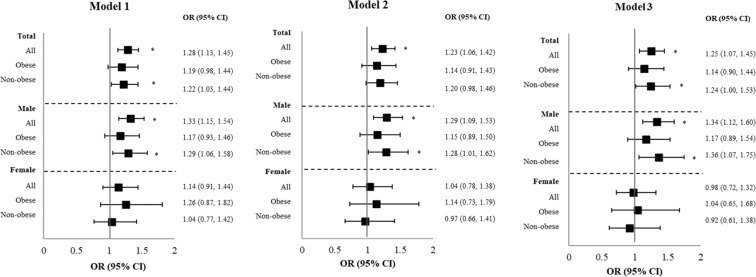
Forest plot of risk estimates for NAFLD and CAC stratified by sex and obesity status. Plots illustrate the odds ratios for identifying the association between CAC and NAFLD stratified by obesity status and sex after adjustment for multiple confounders. *P < 0.05.

The forest plot (Fig. 2) illustrates the stratified analysis of the association between NAFLD and CAC among sex-stratified participants with and without obesity after sequential adjustments. In models 1, 2, and 3, NAFLD was significantly associated with CAC among non-obese male participants (model 1: OR, 1.29 [1.06–1.58], *P* = 0.012; model 2: OR, 1.28 [1.01–1.62], *P* = 0.039; model 3; OR, 1.36 [1.07–1.75], *P* = 0.014). However, there was no significant association between NAFLD and CAC in female participants with or without obesity.

In a mediation analysis with NAFLD acting as a mediator, obesity was associated with CAC in both male and female participants (Supplementary Fig. S1 and Supplementary Table S2).

## Discussion

The present study showed that NAFLD was significantly associated with CAC in a large, health-screening population without previous CVD history after multiple adjustments including those for known CVD risk factors and markers of liver disease. Moreover, in our subgroup analysis, the association between NAFLD and CAC seemed to be affected by sex and obesity status, indicating that NAFLD could be a notably influential risk factor for CAC, especially in non-obese male participants but not in female or obese male participants.

Previous studies showed that NAFLD was significantly associated with adverse CVD events in the general population^32–38^. Independent of traditional cardiovascular and metabolic risk factors, NAFLD has been reported to be associated with CAC^5,8,39–42^, which is closely correlated with the severity of atherosclerosis and directly proportional to increased cardiovascular event rates^13,43^. A recent large longitudinal cohort study demonstrated that NAFLD was significantly associated with the development of CAC independent of time-dependent BMI in participants with no history of CVD^8^. However, the Diabetic Heart Study showed that hepatic steatosis was not significantly correlated with coronary calcium in randomly selected participants, although it was correlated with visceral and subcutaneous fat after adjustment for age, race, sex, BMI, and diabetes status^17^. A study of postmenopausal women showed a significant correlation between NAFLD and the prevalence of CAC, but this association was not statistically significant after adjustment^19^.

Through univariate analysis, the present study showed that NAFLD was significantly associated with CAC when all participants were considered. However, NAFLD showed different associations with CAC according to obesity status after adjustment for multiple risk factors. Notably, a significant association between NAFLD and CAC was only observed in non-obese participants, whereas the same was not true for obese participants.

A previous study reported that the prevalence of non-alcoholic steatosis and fibrosis is not significantly different between non-obese and obese patients with NAFLD. Further, visceral obesity, as opposed to general obesity, may be associated with NAFLD in non-obese patients^12^. Ectopic fat accumulation, such as that in cases with visceral fat, epicardial adipose tissue, or NAFLD, has been proposed as a more important predictor of CVD than general fat accumulation^44^. Previous studies noted that epicardial adipose tissue has a positive correlation with CVD evaluated by non-invasive or invasive angiography and that it had a stronger correlation with CVD than other body fat distribution, especially in non-obese males^45,46^. Although non-obese patients with NAFLD may less frequently have clinically evident metabolic disorders than overweight or obese patients with NAFLD, they had an increased risk for metabolic disorders and should be prospectively evaluated^11,12^. A recent large cohort study using health-screening program data showed that CAC was more closely associated with NAFLD than with abdominal obesity in male participants. Although the mean CAC score was the highest in participants with abdominal obesity without NAFLD, subjects with NAFLD alone showed an increased risk for CAC, whereas subjects with abdominal obesity alone showed an insignificantly increased risk for CAC in multivariate analysis^5^. Our data also indicated that NAFLD was an independent risk factor for CAC in all participants after multiple adjustments for factors, including obesity and abdominal obesity. Moreover, the present study showed that NAFLD could mediate the association between obesity and CAC in both male and female participants.

One of the strengths of this study is that it determined the effects of NAFLD on CAC according to obesity status and sex using a three-way interaction effect and showed that the association between NAFLD and CAC was influenced by sex and obesity status. Therefore, we performed a subgroup analysis that showed that NAFLD was a notably influential risk factor for CAC in non-obese male participants, but not in obese male participants or female participants. These findings suggest that NAFLD may be a more important contributor to CAC in non-obese male populations than in female or obese male populations. Our results suggest that the use of multiple comparisons might be another explanation for the observed difference among subgroups.

The mechanisms underlying the deleterious effects of NAFLD on CVD outcomes are not clear. Insulin resistance, oxidative stress, altered lipoprotein metabolism and adipokine levels, and chronic inflammation have been shown to be associated with the development of accelerated atherosclerosis in patients with NAFLD^5,32,38,47^. Further research is needed to unveil more specific mechanisms by which NAFLD may contribute to the development and progression of atherosclerosis^21^.

This study has several limitations. First, our study was cross-sectional in design. Therefore, the precise relationship between NAFLD and CAC remains unclear based on obesity status. Prospective studies should be performed to determine whether the association between NAFLD and CAC differs according to obesity status. Second, NAFLD was determined using ultrasonography rather than histologic evaluation. However, ultrasonography is widely used in the clinical setting and has been shown to be able to independently detect CVD^38^. Third, the assessments for smoking and alcohol use were based on a self-administered structured questionnaire during a health check-up program. Finally, diagnostic coronary angiography could not be performed to confirm the relationship between NAFLD and the presence of CAC.

In conclusion, NAFLD was significantly associated with CAC in healthy-screening program participants. However, this association appeared to differ according to sex and obesity status. Future studies on NAFLD should focus on non-obese male participants who are most likely to benefit from intensive lifestyle modification and pharmacological treatment to decrease CVD risk.

## Supplementary information

Supplementary Information.

## Data Availability

The datasets analysed during the current study are not publicly available due to current Korean law regulating release of personal information, even in the cases of research purposes. However, limited data may be disclosed only after approval from Institutional Review Board (IRB) of Gangnam Severance Hospital, Yonsei University College of Medicine, Seoul, Korea. Contact information of IRB is “http://gsocr.yuhs.ac/Gangnam/gangnamintro.aspx”.
