# Supplementary material for: Association between non-alcoholic fatty liver disease and coronary calcification depending on sex and obesity
Source: Sci Rep. 2020 Jan 23;10:1025. doi: 10.1038/s41598-020-57894-y (PMC6978333; doi:10.1038/s41598-020-57894-y)
Supplement: Supplementary file 1 — Supplementary Information. [file 41598_2020_57894_MOESM1_ESM.docx]

**Association between non-alcoholic fatty liver disease and coronary calcification depending on sex and obesity**

**Seok-hyung Kim, MD^1^; Hae Yeul Park, MD^2^; Hye Sun Lee, PhD^3^; Kwon Soo Jung, MD^2^; Moon Hyoung Lee, MD^2^; Jong Hyun Jhee, MD^2^; Tae Hoon Kim, MD^4^; Jung Eun Lee, MD, PhD^4^; Hyung Jong Kim, MD, PhD^5^; Beom Seok Kim, MD, PhD^6^; Hyeong Cheon Park, MD, PhD^2, 7^; Byoung Kwon Lee, MD, PhD^2^; Hoon Young Choi, MD, PhD^2, 7^***

**Supplementary Information**

**Contents**

**Table S1. Univariate logistic regression analysis to determine risk factors affecting CAC------------------------------------ 2**

**Table S2. NAFLD as a mediator of the association between obesity and CAC -------------------------------------------------- 3**

**Figure S1. Mediation analysis of the association between obesity and CAC in which NAFLD was considered as a mediator ---------------------------------------------------------------------------------------------------------------------------------------- 4**

**Table S1. Univariate logistic regression analysis to determine risk factors affecting CAC**

| **Variables** | **All participants** | | **Obese participants** | | **Non-obese participants** | |
| --- | --- | --- | --- | --- | --- | --- |
|  | **OR (95% CI)** | ***P*-value** | **OR (95% CI)** | ***P*-value** | **OR (95% CI)** | ***P*-value** |
| Age | 1.11 (1.10–1.11) | <0.001 | 1.09 (1.08–1.10) | <0.001 | 1.12 (1.11–1.13) | <0.001 |
| Male sex | 2.89 (2.58–3.23) | <0.001 | 1.86 (1.55–2.23) | <0.001 | 3.33 (2.88–3.85) | <0.001 |
| Hypertension | 2.54 (2.29–2.82) | <0.001 | 2.08 (1.76–2.45) | <0.001 | 3.29 (2.77–3.90) | <0.001 |
| Diabetes | 3.25 (2.82–3.76) | <0.001 | 2.02 (1.49–2.73) | <0.001 | 3.38 (2.57–4.43) | <0.001 |
| Obesity | 1.78 (1.61–1.97) | <0.001 |  |  |  |  |
| Abdominal obesity | 3.90 (3.44–4.42) | <0.001 | 4.05 (2.68–6.14) | <0.001 | 3.81 (3.30–4.41) | <0.001 |
| NAFLD | 1.83 (1.65–2.02) | <0.001 | 1.24 (1.05–1.46) | 0.011 | 1.88 (1.63–2.16) | <0.001 |
| LDL-cholesterol | 0.99 (0.99–1.00) | 0.259 | 1.00 (1.00–1.00) | 0.013 | 1.00 (1.00–1.00) | 0.855 |
| eGFR | 0.97 (0.96–0.97) | <0.001 | 0.98 (0.97–0.98) | <0.001 | 0.97 (0.96–0.97) | <0.001 |
| CRP | 1.02 (1.01–1.03) | 0.001 | 1.00 (0.99–1.02) | 0.508 | 1.04 (1.02–1.06) | 0.001 |
| Current smoking | 1.21 (1.05–1.36) | 0.009 | 0.88 (0.71–1.09) | 0.250 | 1.52 (1.25–1.84) | <0.001 |
| Alcohol status | 1.20 (1.07–1.36) | 0.003 | 1.16 (0.96–1.39) | 0.128 | 1.14 (0.97–1.34) | 0.118 |
| AST | 1.01 (1.01–1.02) | <0.001 | 1.01 (1.01–1.02) | <0.001 | 1.01 (1.01–1.01) | <0.001 |
| ALT | 1.01 (1.00–1.01) | <0.001 | 1.00 (1.00–1.01) | 0.384 | 1.01 (1.01–1.02) | <0.001 |
| γ-GT | 1.01 (1.00–1.01) | <0.001 | 1.00 (1.00–1.00) | 0.129 | 1.01 (1.00–1.01) | <0.001 |

CAC, coronary artery calcification; NAFLD, non-alcoholic fatty liver disease; LDL, low-density lipoprotein; eGFR, estimated glomerular filtration rate; CRP, C-reactive protein; AST, aspartate aminotransferase; ALT, alanine aminotransferase; γ-GT, gamma-glutamyl transpeptidase; OR, odds ratio; CI, confidence interval.

**Table S2. NAFLD as a mediator of the association between obesity and CAC**

| **Variables** | **Total (*n*=7272)** | | **Male (*n*=4317)** | | **Female (*n*=2955)** | |
| --- | --- | --- | --- | --- | --- | --- |
|  | **Comparable coefficient (SE)** | ***P*-value** | **Comparable coefficient (SE)** | ***P*-value** | **Comparable coefficient (SE)** | ***P*-value** |
| **Model without mediator** |  |  |  |  |  |  |
| Obesity status 🡪 CAC (c) | 0.152 (0.014) | <0.001 | 0.062 (0.017) | <0.0001 | 0.191 (0.024) | <0.001 |
| **Model with mediator (NAFLD)** |  |  |  |  |  |  |
| Obesity status 🡪 NAFLD (a) | 0.404 (0.013) | <0.001 | 0.364 (0.017) | <0.0001 | 0.383 (0.020) | <0.001 |
| NAFLD 🡪 CAC (b) | 0.123 (0.015) | <0.001 | 0.069 (0.018) | <0.0001 | 0.113 (0.027) | <0.001 |
| Obesity status 🡪 CAC (c’) | 0.106 (0.015) | <0.001 | 0.039 (0.018) | <0.0001 | 0.150 (0.026) | <0.001 |
| Indirect effect (a * b) | 0.050 (0.006) | <0.001 | 0.025 (0.007) | 0.0002 | 0.043 (0.010) | <0.001 |
| **Baron and Kenny’s percentage (proportion) of effect mediated** | 32.014% | | 39.295% | | 22.46% | |

CAC, coronary artery calcification; NAFLD, non-alcoholic fatty liver disease.

**Figure S1. Mediation analysis of the association between obesity and CAC in which NAFLD was considered as a mediator**

**
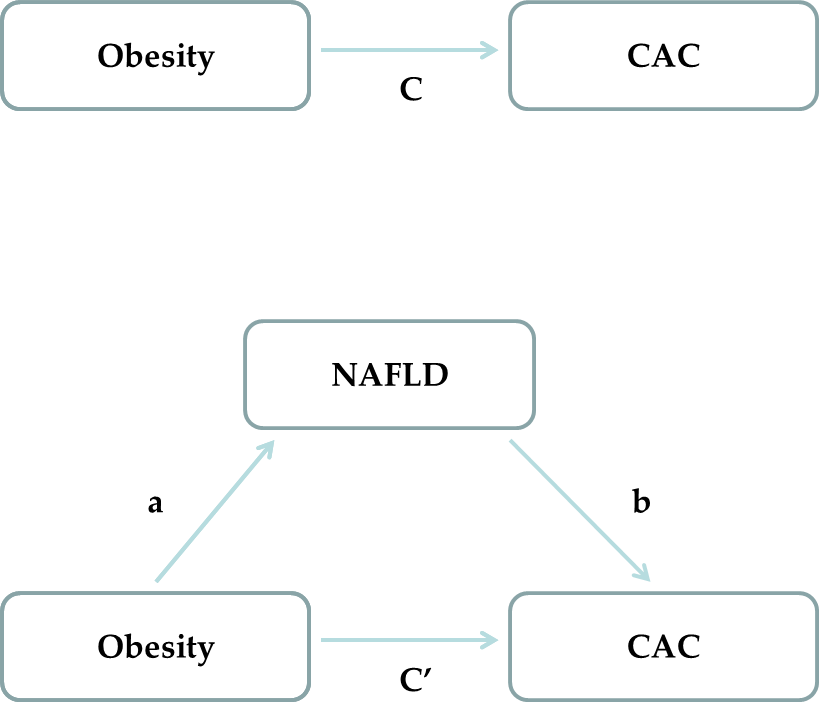
**

Pathway C represents the model without mediator. Pathway a * b represents the pathway mediated by NAFLD. Pathway C’ represents the pathway not mediated by NAFLD. AC, coronary artery calcification; NAFLD, non-alcoholic fatty liver disease.
